# Demonstration of Metabolic and Cellular Effects of Portal Vein Ligation Using Multi-Modal PET/MRI Measurements in Healthy Rat Liver

**DOI:** 10.1371/journal.pone.0090760

**Published:** 2014-03-05

**Authors:** András Fülöp, Attila Szijártó, László Harsányi, András Budai, Damján Pekli, Diána Korsós, Ildikó Horváth, Noémi Kovács, Kinga Karlinger, Domokos Máthé, Krisztián Szigeti

**Affiliations:** 1 1st Department of Surgery, Semmelweis University, Budapest, Hungary; 2 Department of Biophysics and Radiation Biology, Semmelweis University, Budapest, Hungary; 3 CROmed Translational Research Centers, Budapest, Hungary; 4 Department of Radiology and Oncotherapy, Semmelweis University, Budapest, Hungary; The Chinese University of Hong Kong, Hong Kong

## Abstract

**Objectives:**

In the early recognition of portal vein ligation (PVL) induced tumor progression, positron emission tomography and magnetic resonance imaging (PET/MRI) could improve diagnostic accuracy of conventionally used methods. It is unknown how PVL affects metabolic patterns of tumor free hepatic tissues. The aim of this preliminary study is to evaluate the effect of PVL on glucose metabolism, using PET/MRI imaging in healthy rat liver.

**Materials and Methods:**

Male Wistar rats (n = 30) underwent PVL. 2-deoxy-2-(^18^F)fluoro-D-glucose (FDG) PET/MRI imaging (nanoScan PET/MRI) and morphological/histological examination were performed before (Day 0) and 1, 2, 3, and 7 days after PVL. Dynamic PET data were collected and the standardized uptake values (SUV) for ligated and non-ligated liver lobes were calculated in relation to cardiac left ventricle (SUV_VOI_/SUV_CLV_) and mean liver SUV (SUV_VOI_/SUV_Liver_).

**Results:**

PVL induced atrophy of ligated lobes, while non-ligated liver tissue showed compensatory hypertrophy. Dynamic PET scan revealed altered FDG kinetics in both ligated and non-ligated liver lobes. SUV_VOI_/SUV_CLV_ significantly increased in both groups of lobes, with a maximal value at the 2^nd^ postoperative day and returned near to the baseline 7 days after the ligation. After PVL, ligated liver lobes showed significantly higher tracer uptake compared to the non-ligated lobes (significantly higher SUV_VOI_/SUV_Liver_ values were observed at postoperative day 1, 2 and 3). The homogenous tracer biodistribution observed before PVL reappeared by 7^th^ postoperative day.

**Conclusion:**

The observed alterations in FDG uptake dynamics should be taken into account during the assessment of PET data until the PVL induced atrophic and regenerative processes are completed.

## Introduction

Despite advances in chemotherapy regimens and ablative techniques, extended hepatectomy is considered as a gold standard for the treatment of primary and secondary liver cancers. [1]–[3] Nevertheless, surgical resection can be accomplished only in 20–30% of the cases, due to insufficient amount of remaining liver mass after hepatectomy (future remnant liver volume; FRLV). [4] It is known for decades that occlusion of the portal vein results in atrophy of the occluded liver lobe, while owing to the unique regeneration capacity, the unoccluded liver tissue shows compensatory hypertrophy. [5] Based on this observation portal vein occlusion techniques, such as portal vein ligation (PVL) or embolization (PVE), have been introduced to increase the future volume of the remnant liver before major liver resection. [6] Considering that at the time of diagnosis most liver malignancies show multiple bilateral patterns, a two-stage resection with portal vein occlusion has recently been developed. During the first stage of this novel treatment procedure, small metastasectomies in the potential future remnant liver in combination with the occlusion of the contralateral portal vein are performed with or without chemotherapy. In the second curative stage, after compensatory hypertrophy of cleared unoccluded lobes, the portal-deprived liver lobes with residual tumors are removed. [7].

Although, the above detailed two-stage procedure is more widely used, there is increasing evidence that PVL/PVE-induced changes have a strong stimulatory effect on tumor growth as well. Thus, there might be an increased risk, that portal vein occlusion could stimulate not only the growth of future remnant liver, but also significantly affects tumor size and tumor spread in both occluded and non-occluded liver segments. [8] Several clinical and preclinical studies have demonstrated the increased proliferative activity of malignant cells after preoperative portal vein occlusion. [9]–[11] Tumor progression in the liver between the time of PVL/PVE and the next phase of hepatectomy is responsible for the majority of changes into irresectable state which occurs in approximately 20% of the cases of planned for two-stage surgeries. [12], [13].

The average time to achieve optimal FRLV (optimal time of the second, curative operation) is approximately 1 month after PVL/PVE. [14] During this waiting period, the monitoring of regenerative process and tumor progression are essential. In a clinical scenario, computed tomography (CT) is currently the gold-standard method of assessing the increase in FRLV. [15] However, CT volumetry may not be reliable in the detection of residual tumor growth, because the accuracy of CT is poor for smaller (<0.2–1 cm) tumor sizes. [16] MRI, especially with liver-specific contrast agents, could improve the sensitivity by providing better soft-tissue contrast, but is not sufficiently specific leading to number of false positive lesions. [17] These drawbacks of morphological imaging techniques indicate the importance of additional functional imaging assays, which could more specifically recognize tumor progression during two-stage hepatectomy combined with portal vein ligation.

In clinical oncology positron emission tomography (PET) is an extremely valuable tool for tumor diagnosis, staging, restaging and for treatment response follow-up. [18] To image hepatic neoplasia several PET radiopharmaceuticals are available, such as [^11^C]-acetate, [^11^C]-choline and 2-deoxy-2-(^18^F)fluoro-D-glucose (FDG). In the oncologic use of PET, however, FDG is the most commonly used tracer, which is able to assess the typically enhanced glucose transport and metabolism in malignant tumors. FDG was found to be superior in detecting residual tumor growth and tumor recurrence compared to morphologic imaging procedures, furthermore, FDG-PET is the primary imaging modality for detection of distant (extrahepatic) metastasis. [19] Although, FDG is an excellent tracer in case of tumor localization, it is not tumor specific. [20] Increased FDG uptake was observed in several energy-intensive processes such as inflammation, as well as during regeneration. The regenerative and atrophic process induced by the ligation/embolization of portal vein is associated with not only volumetric, but also with hemodynamic and metabolic changes. [21] These complex alterations after PVL/PVE may also affect the FDG uptake patterns of tumor free hepatic tissues, which constitute a challenge in the differentiation of these processes from intrahepatic tumor spread. Therefore, unfolding the changes regarding FDG uptake after PVL/PVE in a tumor free, healthy liver is essential in the early recognition of tumor progression.

There is no study in the literature that assesses the changes in FDG uptake pattern of normal liver after PVL/PVE. Kinetic FDG data combined with volumetric information available from MR imaging of the liver, are more reliable than PET alone or PET/CT. Thus, the aim of our present study was to selectively evaluate dynamics of FDG uptake in occluded and non-occluded liver lobes by in vivo multimodal FDG PET-MRI (nanoScan PET/MRI) in a well-established rat model of portal vein ligation.

## Materials and Methods

### Portal Vein Ligation (PVL)

The experimental design was regulated in strict adherence to the National Institutes of Health guidelines for animal care and approved by Committee on Animal Experimentation of Semmelweis University (permit number: 22.1/2408/3/2011 and XIV-I-001/29-7/2012). 200–250 g male Wistar rats (Charles River Hungary Ltd., Budapest, Hungary) were kept on standard chow and water *ad libitum* in specific, pathogen-free conditions at 22–24°C with a 12-hour light-dark cycles. Animals were allowed to acclimatize for 1 week before starting experiments. After intra-peritoneal injection of ketamine (75 mg/kg) and xylazine (7.5 mg/kg), animals were placed in a supine position and median laparotomy was performed. The median and left lateral lobes were gently mobilized to reveal the portal pedicels. Under operating microscope (Zeiss Opmi Surgical Microscope, Carl Zeiss Meditec AG, Jena, Germany) the portal branches feeding the median, left lateral and caudate lobes (approximately 80% of total liver mass) were dissected and completely ligated with 6–0 silk. Great care was taken to avoid damaging the vulnerable hepatic artery and bile duct. At the end of the procedure the liver lobes were gently repositioned anatomically, the peritoneal cavity was closed in two layers by running suture (4–0) and the animals were returned to their cages until further examination. Before (0 day) and 1, 2, 3, 7 days after portal vein ligation, rats were sacrificed by exsanguination for histological analysis. The liver was removed and the ligated and non-ligated lobes were weighted separately after expulsion of blood by gentle compression with gauze. Liver specimens were taken from the excised left lateral lobe (ligated lobe) and the superior right lobe (non-ligated lobe). Samples were fixed in 4% neutral-buffered formalin and embedded in paraffin for further histological and immunohistochemical examination (see below). At each time point, six animals were used (n = 6; in all n = 30).

### Positron Emission Tomography/Magnetic Resonance Imaging (PET/MRI)

In a separate study additional 6 animals underwent imaging 12 hours before and 1, 2, 3 and 7 days after portal vein ligation. Images were acquired with a PET/MRI (nanoScan PET/MRI, Mediso Ltd., Hungary) sequential animal imaging system. Blood glucose levels were confirmed to be within the normal range before injection of the radioactive tracer and throughout the study. In the PET experiment 6.5±1.0 MBq of FDG in 0.2 mL volume was injected into the tail vein, under anesthesia (2% isoflurane in 100% O_2_ gas). To prevent movement, the animals were fixed to a rat chamber (MultiCell Imaging Chamber, Mediso Ltd., Hungary) and positioned in the center of field of view (FOV). Dynamic list-mode PET data were collected in three-dimensional acquisition mode. The MRI images (matrix size 140×140; 0.5 mm; 30 slices; 1.3 mm; gap 0.5 mm) were acquired for 50 minutes (25 minutes for each imaging) with T1-weighted (SE2D; TR/TE 800/10 ms; FOV 70 mm; NEX 4), T2-weighted (FSE2D; TR/TE 4733/60 ms; FOV 70 mm; NEX 2) sequences. PET volumes were reconstructed using a three-dimensional Ordered Subsets Expectation Maximization (3D-OSEM) algorithm (Tera-Tomo, Mediso Ltd., Hungary) into dynamic frames of 10×30 s, 10×60 s, 10×270 s (total 60 min, 30 frames) for all scans with a voxel size of 0.3 mm. Static images were generated based on frame 21 (15^th^ to 19^th^ minutes were integrated after tracer injection). PET and MRI images were automatically coregistered by the PET/MRI instrument’s acquisition software. Reconstructed, reoriented and coregistered images were further analyzed with Fusion (Mediso Ltd., Hungary) and VivoQuant (inviCRO LLC, US) dedicated image analysis softwares by placing appropriate Volume of Interests (VOI) on the organs. Two volume of interests were delineated manually slice by slice on each T1 weighted scans. One VOI consisted of the ligated liver lobes and the other of the non-ligated liver lobes. For volumetric analysis the built-in VOI statistics of the VivoQuant software was used, which determines the volume from voxel size and number of voxels in the VOIs. In order to get more precise values of the volumes, the T1 weighted slices were interpolated through the slice direction to create an isovoxel data set. For FGD uptake, the standardized uptake value (SUV) was calculated by dividing the mean radioactivity concentration (MBq/mL) by injected dose per body weight (MBq/g). The mean SUV for ligated and for non-ligated liver lobes were selectively recorded. The mean SUV of corresponding liver lobes was expressed in relation to a reference tissue SUV (cardiac left ventricle; SUV_VOI_/SUV_CLV_) and to the mean liver SUV (SUV_VOI_/SUV_Liver_).

### Evaluation of Liver Regeneration and Atrophy

Liver lobe wet weight was measured separately after exsanguinations using a laboratory scale (Mettler Toledo AG 245, Mettler-Toledo LLC, Columbus, OH, readability: 0.01 mg/0.1 mg). The relative weight of ligated and non-ligated lobes to the body weight (BW) was then calculated and expressed as follows: liver weight (g)/100 g BW. The liver volume change was measured by MRI and the relative liver volume was expressed as mm^3^/100 g BW. The change in liver volume calculated from PET/MRI was compared with the results derived from autopsy.

### Histological and Immunohistochemical Examination

After liver weight measurement, one slice was removed for pathological examination from the same anatomic position in each animal. The formalin fixed, paraffin embedded 3–5 micrometer thick sections underwent light microscopic observation after hematoxylin and eosin staining. *Necrosis* was assessed by morphological features (swollen cytoplasm, vacuolization, disrupted cell and organelle membranes and lytic nuclear changes) on each section. The extent of necrosis was graded on a scale of 0–4 described by Suzuki [22]: grade 0– absence of necrosis; grade 1– single cell necrosis; grade 2–30% lobular necrosis; grade 3–60% lobular necrosis; grade 4– more than 60% lobular necrosis. Hepatocyte *apoptotic cell death* was identified also by morphological criteria (cell shrinkage, chromatin condensation, margination and apoptotic bodies) and was counted in 10 consecutive high-power fields (HPF, x200) and expressed in absolute numbers.

For the detection of *mitotic activity* Ki-67 immunohistochemical staining was applied on formalin-fixed, paraffin-embedded tissues. Avidin–biotin–peroxidase complex immunohistochemical method was performed for antigen retrieval. [23] Primary antibodies were MIB-5 antibody against Ki-67 (DAKO, Glostrup, Denmark) of hepatocytes. Labeling index was defined as the mean number of Ki-67-positive cells per HPF (×200), respectively.

To determine *cellular glycogen content*, periodic acid and Schiff (PAS) staining was used. The intensity of PAS staining was graded on a five tier scale (0: no staining –5: highly intense). The level of glycogen content was determined by scoring at least three independent microscopic fields for each sample.

The above mentioned histological and immunohistochemical evaluations were performed in blinded fashion by two experienced pathologists.

### Statistical Analysis

Mann–Whitney U test and two sample Student t-test were used to compare data between groups. Normality was tested by Kolmogorov-Smirnov test. Correlation between variables was tested by Spearman’s method. All tests were performed using the Statistical Package for Social Sciences (IBM SPSS Statistics 20.0 software, IBM Corporation, Armonk, NY, USA). Values are expressed as means ± S.D. A p<0.05 confidence interval was considered as statistically significant.

## Results

### Liver Hypertrophy and Atrophy Induced by Portal Vein Ligation

**Figure 1:**
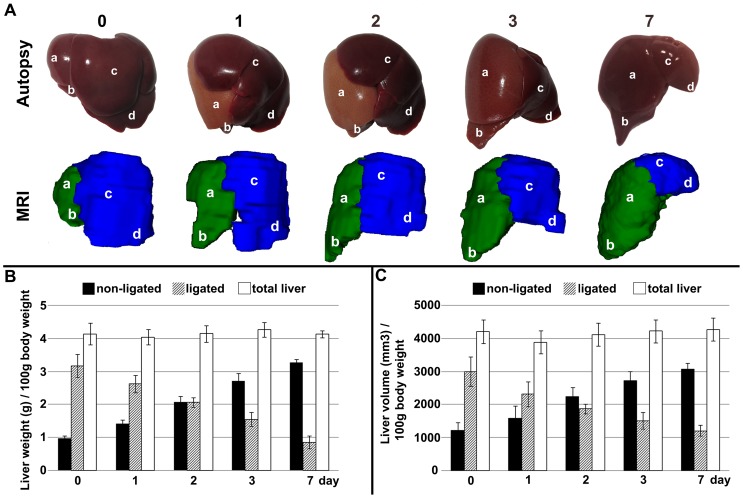
Changes in weight and volume of the liver after portal vein ligation. A: The macroscopic appearance of liver by post mortem examinations and three dimensional MRI reconstruction preoperatively (0), as well as 1, 2, 3 and 7 days after portal vein ligation (PVL). Changes in weight (B) and volume (C) of total liver, ligated and non-ligated liver lobes at the examined time points. The alteration in liver weight showed a significant positive correlation with the volume change (r = 0.842, P<0.001; tested by Spearman’s method). Values are expressed as mean ± S.D. Each column represents the average of 6 animals. a =  superior right lateral lobe; b =  inferior right lateral lobe; c =  median lobe; d =  left lateral lobe.

The weight of the portal-deprived lobes decreased rapidly after the operation and reached approximately one-fourth its original weight on postoperative day 7. On the other hand the non-ligated liver lobes underwent compensatory gain in weight. By a balance of atrophy and hypertrophy, the weight of total liver was maintained near to the baseline throughout the entire experiment. The volume of liver lobes changed in parallel with the liver weights. The volume change, based on MRI volumetry, correlated well with the weight change derived from autopsy (r = 0.842; p<0.001); (Figure 1.).

### Histomorphological Characteristics of Ligated and Non-ligated Liver Lobes

**Figure 2:**
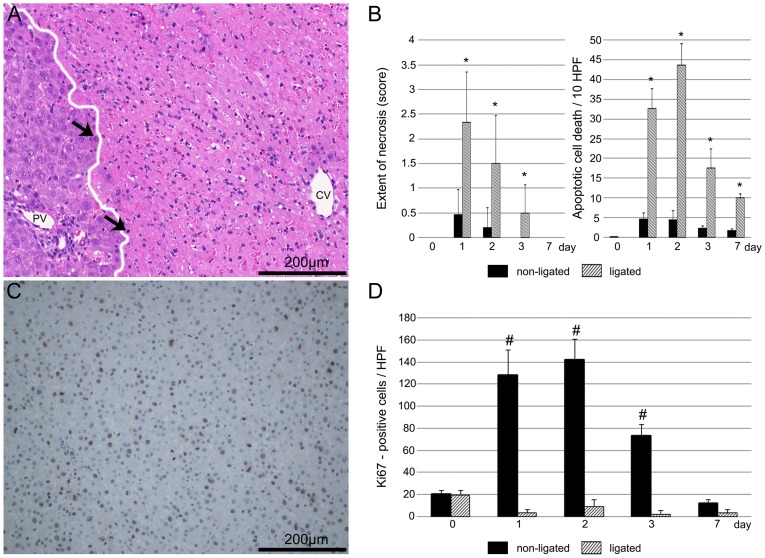
Histomorphological characteristics of the liver after portal vein ligation. A: Histomorphological characteristics of the ligated lobes 1 day after portal vein ligation (PVL). Wide necrotic areas were seen around the central vein. At the boundary between the necrotic and non-necrotic areas (white line) apoptosis was frequently present (black arrows). B: The extent of necrotic areas and the number of apoptotic cell death significantly increased in ligated lobes compared with that seen in non-ligated liver lobes. C: Ki-67 labeling in non-ligated lobes on 2^nd^ day after surgery. Ki-67 antibody reacts with the nuclear structure present exclusively in the proliferative phase of the cell cycle. In the non-ligated liver lobes immunohistochemistry (brown stain) show a high expression level of Ki-67 in cell nuclei, which represent an increased mitotic activity of the hepatocytes. D: Mitotic activity of hepatocytes significantly increased in non-ligated liver lobes, with the peak response on day 2. In contrast, the Ki-67 positivity of ligated lobes did not change significantly throughout the experiment. Values are expressed as mean ± S.D. Each column presents the average of 6 animals. PV =  portal vein; CV =  central vein; * =  p<0.05 versus non-ligated lobes; # =  p<0.05 versus ligated lobes.

Atrophy of portal-deprived liver lobes was mainly caused by the combination of necrosis and apoptosis. Extensive coagulation necrosis occurred in the central zone of liver lobules one day after the ligation. The necrotic areas became significantly reduced in size at postoperative day 2 and 3. At this time, a high number of macrophages were observed in the necrotic region. Seven days after the operation necrosis could not be observed, while the normal hepatic architecture recovered, with no signs of fibrosis. The number of apoptotic hepatocytes also increased rapidly after the operation and reached the peak on postoperative day 2. Apoptosis was most frequently present at the boundary between necrotic and non-necrotic areas and remained elevated until the 7^th^ postoperative day. In contrast, in the non-ligated liver lobes, the presence of necrosis and apoptosis was not typical (Figure 2. A–B).

To examine proliferating capacity of the liver Ki-67 immunohistochemical staining was performed. Ki-67 is expressed by proliferating cells in late G1, S, G2 and M phases, however it is absent in the resting, G0 phase, therefore it is a reliable marker to detect mitotic activity. With this immunohistochemical staining, increased mitotic activity was found in non-ligated lobes with the peak response at 2^nd^ days. Thereafter, the number of positive cells decreased and reached the baseline level at 7^th^ postoperative day. However, in the ligated liver lobes the number of Ki-67 positive cells did not change significantly during the experiment (Figure 2. C–D).

**Figure 3:**
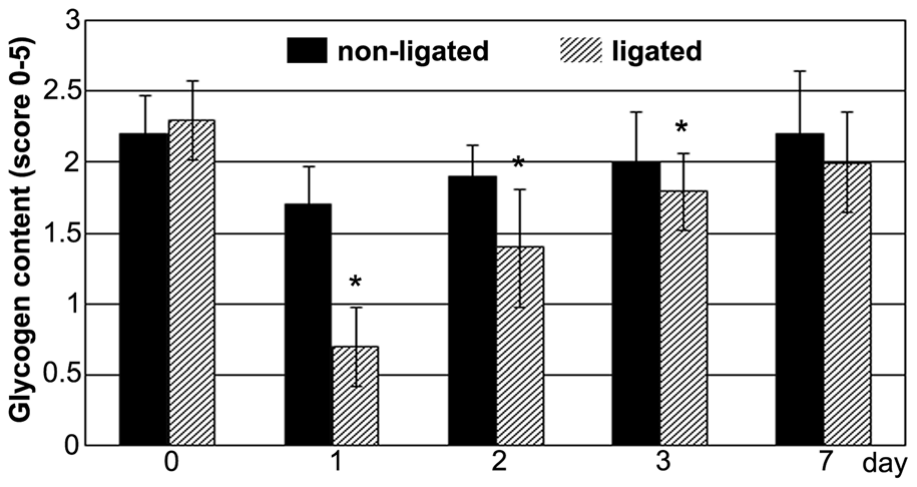
Glycogen content of cells. Glycogen content of ligated lobes (striped column) decreased markedly after portal vein ligation and it reached the baseline level at postoperative day 7. In non-ligated lobes (black column), the PAS staining did not show any significant alteration throughout the experiment. Values are expressed as mean ± S.D. Each column represents the average of 6 animals. * =  p<0.05 versus the baseline level (0 day) of the corresponding lobes.

Liver showed a moderate, homogeneous glycogen staining before (day 0) PVL. Day 1 after ligation, PAS positive glycogen granules almost completely disappeared in ligated lobes and the glycogen content of cells returned to baseline level just at day 7. In contrast, the PAS staining in non-ligated liver lobes did not show significant alterations compared to the baseline level (day 0) in any examined time point (Figure 3).

### ^18^F-FDG Data Analysis

**Figure 4:**
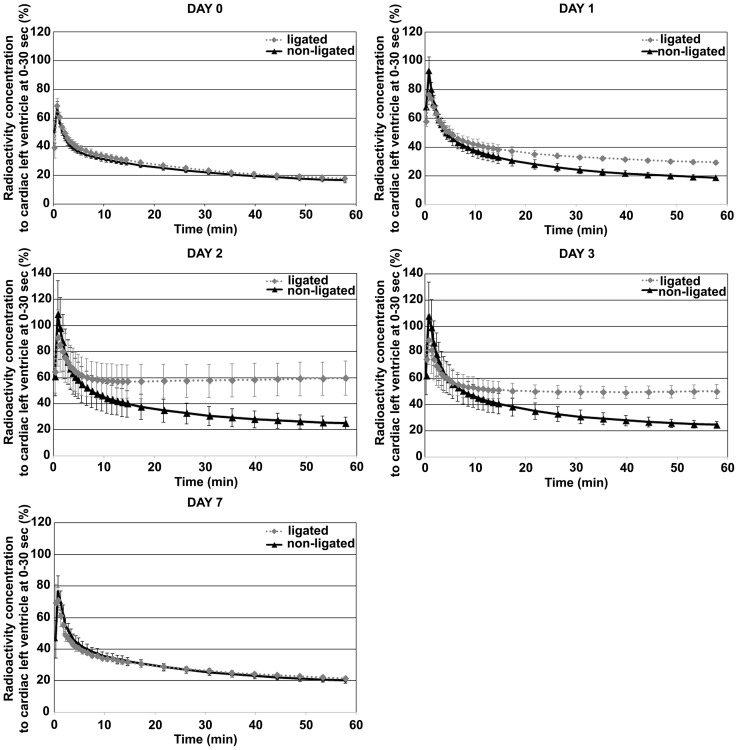
Time activity curves of ligated and non-ligated lobes at different time points. After portal vein ligation (PVL) the characteristics of curves showed altered tracer kinetics in both ligated (gray; dotted line) and non-ligated (black; solid line) liver lobes, but these changes in ligated lobes were more pronounced. The most significant alterations can be seen on 2^nd^ postoperative day, then 7 days after the operation the curves characteristic showed similar appearance to the pre-PVL data.

**Figure 5:**
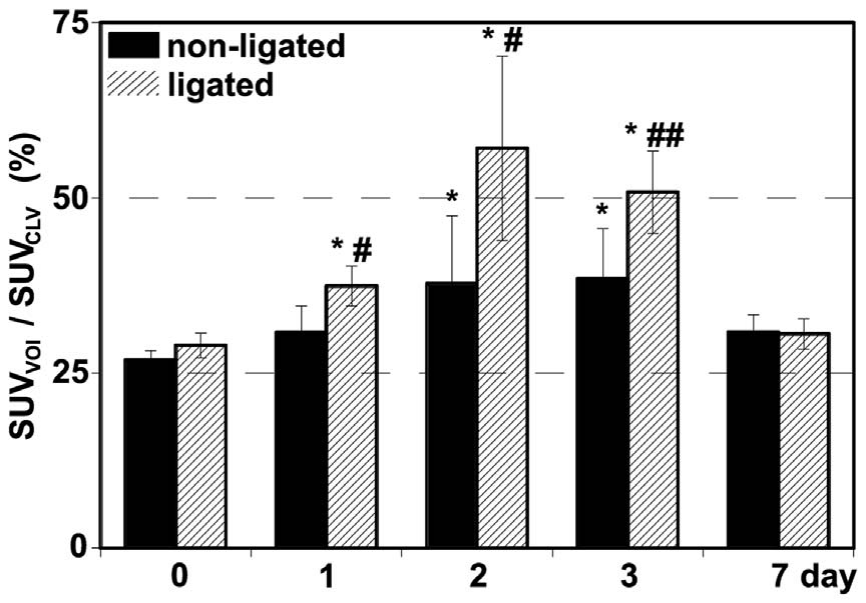
The ratio of mean SUV of ligated and non-ligated lobes to cardiac left ventricle SUV. The FDG uptake significantly increased in ligated (striped column) liver lobes, with maximal value on 2^nd^ postoperative day and returned to near to the baseline 7 day after portal vein ligation. In non-ligated (black column) lobes the tracer uptake also increased, but significantly lower extent compared to the ligated lobes. Values are expressed as mean ± S.D. Each column represents the average of 6 animals. * =  p<0.05 versus the baseline level (0 day) of the corresponding lobes; # =  p<0.05 versus non-ligated lobes; ## =  p<0.01 versus non-ligated lobes.

**Figure 6:**
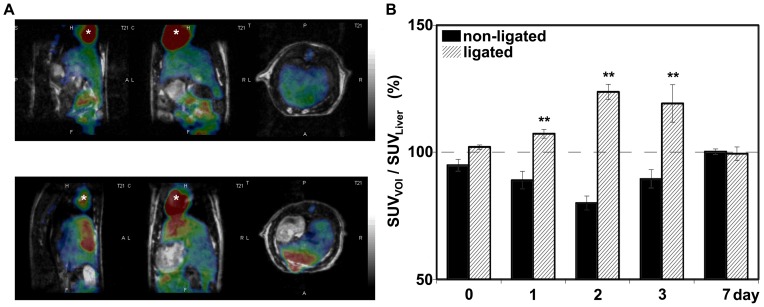
Tracer biodistribution in ligated and non-ligated liver lobes. A:Representative coregistered PET and MRI image of rat liver before (top) and 3 days after (bottom) portal vein ligation (PVL). The PET reconstruction is based on frame 21 (at 15–19 minutes). The top inset picture shows a homogeneous liver FDG uptake pattern in both liver lobes. The small inhomogeneity is related to the morphology of liver. After PVL the liver shows inhomogeneous tracer distribution with significantly increased FDG uptake in ligated lobes, while the tracer uptake in cardiac left ventricle (*) remain constant. B: The ratio of mean SUV of ligated (striped column) and non-ligated (black column) liver lobes to mean liver SUV. The SUV_VOI_/SUV_Liver_ significantly increased in ligated liver lobes compared to the non-ligated lobes after PVL with the peak response on day 2. Values are expressed as mean ± S.D. Each column represents the average of 6 animals. ** =  p<0.01 versus non-ligated lobes.

Dynamic PET scans revealed an altered FDG kinetics characterized by prolonged tracer elimination in both ligated and non-ligated liver lobes. The alterations in time activity curves of ligated lobes were more remarkable compared to the non-ligated lobes (Figure 4.). Before PVL the liver showed homogenous tracer biodistribution. After PVL, FDG uptake within the liver shifted towards the ligated lobes. The SUV_VOI_/SUV_CLV_ showed a significantly increased FDG uptake in ligated liver lobes. That value was maximal on 2^nd^ postoperative day and returned near to the baseline at the 7^th^ day. In non-ligated lobes the SUV_VOI_/SUV_CLV_ also increased, but in a significantly lower extent compared to the ligated lobes. The increment in FDG uptake of non-ligated lobes reached a statistically significant level on 2^nd^ and 3^rd^ postoperative day only, as compared to the baseline (Figure 5.). SUV_VOI_/SUV_Liver_ in ligated lobes significantly increased compared to that in non-ligated liver lobes with the maximal level on 2^nd^ postoperative day. Then at 7^th^ postoperative day the SUV_VOI_/SUV_Liver_ returned to baseline levels and the lobes of liver showed again similar FDG uptake pattern (Figure 6. A–B).

## Discussion

In the recent years two-stage hepatectomy with portal vein ligation became widely used to surgically treat large primary or secondary liver tumors. However portal vein occlusion not only induces liver regeneration, but also potentially results in loco-regional or metastatic tumor spread, making liver resection difficult. [9] In the early recognition of postoperative tumor progression (regional and distant metastases alike), the anatomic information derived from conventional CT or MRI can be insufficient, thus the introduction of functional imaging assays is required to improve diagnostic accuracy. However, to draw adequate conclusion from PET studies, exploring the alteration in tracer kinetics in normal, tumor free liver tissues after PVL is necessary.

In our experiment PET/MRI was used to evaluate the PVL induced alterations in the FDG uptake in parallel with the change in volume of ligated and non-ligated liver lobes in healthy rats. In our study, PVL induced volume and weight changes in liver mass, that are typical in such cases. [5] The ligated liver lobes underwent a massive atrophic process, while the non-ligated lobes showed compensatory hyperplasia. As a result the total liver mass was unchanged throughout the entire experiment. This progressive volumetric alteration was completed by postoperative day 7.

In parallel to the volume changes, dynamic PET data revealed altered FDG kinetics in both the ligated and non-ligated liver lobes after PVL. To accurately determine the alteration in FDG uptake, mean SUV of liver lobes were compared to a reference image derived from the blood input of the left cardiac ventricle. SUV_VOI_/SUV_CLV_ highly significantly increased in ligated lobes, while in the non-ligated lobes just a moderate increase was observed. The different FDG uptake of the ligated and non-ligated lobes was more obvious, when the SUV of corresponding lobes was compared to the whole liver radioactivity concentration. Before PVL, the liver showed homogenous FDG distribution. After PVL, the SUV_VOI_/SUV_Liver_ significantly elevated in the ligated lobes compared to that in the non-ligated part of liver. The contrast between the lobes was the most significant at postoperative day 2. Thereafter, by the postoperative 7^th^ day, when the PVL induced volumetric alterations were completed, the pre-PVL FDG uptake pattern returned and the lobes of liver showed again similar tracer biodistribution.

To explore the cause of these altered FDG uptake pattern, histological examinations were performed. Our results suggest that two main processes could be responsible for the remarkable elevated FDG accumulation in ligated lobes:

1. The atrophy of portal deprived liver lobes is mainly caused by coagulation necrosis, as a result of the cessation of portal vascular inflow and the consequent hypoxic condition around the central vein. [24] The reduction in the numbers of the viable cells through necrosis should be reflected by decreased FDG uptake. [25] However, in the ligated lobes increased FDG accumulation was detected, for which some energy-intensive processes surrounding these necrotic lesions may responsible. (A) *Apoptosis:* At the boundary between necrotic and non-necrotic areas, a large number of apoptotic cells were found. It is well-documented, that apoptosis is an energy-intensive process, because adenosine-triphosphate (ATP) is required for the activation of the efferent molecules of caspase family. However, in these areas around the necrotic fields, a moderate degree of hypoxia is still present. [26] The limited ability of oxygen leads to anaerobic glycolysis, a less efficient use of glucose, with increased necessity of glucose extraction from blood, which consequently could lead to enhanced FDG uptake of the apoptotic cells, and could be reflected in the PET scan. [27]–[29] (B) *Inflammation*: Besides apoptosis, a massive inflammatory reaction was also present around the necrotic regions in the slides. At postoperative day one, macrophages appeared at the rim of the necrotic areas and began to remove the debris. Activated macrophages are characterized with high glucose turnover and consequently increased FDG uptake. [30] It should be noted that, the above detailed histological alterations showed considerable individual variation on first 3 postoperative days, when the atrophic process was the most intensive. This variability also reflected in PET data, which is characterized by a significant deviation during this period.
2. The decreased FDG release from hepatocytes- caused by the elevated glycogenolysis – can also contribute to the increased FDG accumulation in the ligated liver lobes. FDG is a glucose analog, which is normally trapped inside the cells after phosphorylation as FDG-6-phosphate. However, in the liver, FDG-6-phosphate is dephosphorylated and rapidly released, presumably due to the high activity of glucose-6-phosphatase (G-6-Pase). [31] In our experiment an abrupt loss of liver glycogen was observed in the portal deprived liver lobes, which suggests that the ligated lobes may take part in maintaining blood glucose level by accelerated glycogenolysis after PVL. Although, the extent of glycogenolysis is elevated after PVL, the enzyme activity of G-6-Pase of ligated liver lobes are shown to be unaltered. [32] [33] The elevated glycogenolysis and the unchanged G-6-Pase activity of the ligated liver lobes, might result in the intracellular accumulation of glucose-6-phosphate, which is capable to inhibit the dephosphorylation, thereby the release of FDG-6-phosphate by competing for the catalytic unit of G-6-Pase.

In non-ligated liver lobes FDG uptake also increased, but to a significantly lower extent than in ligated lobes, despite the presence of massive regeneration characterized by high mitotic activity of cells. Cell division would supposedly be associated with high glucose/FDG-uptake. [34] However, literature data demonstrated that glucose is not the primary energy source of regeneration in the liver. Already in early phase of regeneration the hepatic metabolism is shifted from carbohydrates utilization to the consumption of lipids. [35] Consistent with this, Brinkmann et al showed that after partial hepatectomy intracellular concentrations of glucokinase decreased to less than one third within the first 72 hours. [36] Given that the level of active glucokinase is a determining factor in FDG uptake, the altered glycolytic activity of hepatocytes during liver regeneration could explain why FDG uptake increased just moderate level in non-ligated liver lobes, despite the significant mitotic activity. Another explanation for moderate FDG uptake in the non-ligated lobes might lie in the high portal glucose supply of liver. Rocheleau et al showed a 230% increase in flow of non-ligated lobes after PVL and 96% of this flow is derived from the portal vein. [37] Taking into account that the portal vasculature receives three times more glucose per minute than the hepatic artery, the glucose supply of the regenerating liver mass is abundant. [38] However, the high plasma glucose levels can inhibit FDG uptake by competing for the binding site of the hepatic glucose transporter proteins. [39] This competition between plasma glucose and FDG could result in the moderate-level of FDG uptake observed in non-ligated, regenerating liver lobes by PET scan.

The above detailed alteration in FDG uptake pattern could have a significant impact on the evaluation of PET studies after PVL. The increased FDG accumulation, especially in the case of ligated lobes, could make difficult the assessment of tumor progression by increased background activity until the PVL induced alterations are completed. However, it also should be noted that, the alteration in FDG uptake of non-ligated lobes was moderate and reached significant level only at the 2^nd^ and 3^rd^ postoperative days. This lower-grade lobar FDG activity might make possible the early recognition of residual tumorous lesions in non-ligated liver shortly after the operation. Considering that, in terms of treatment decision, the critical points are the tumor progression in the future remnant liver (non-ligated lobes) or the formation of distant metastases, the use of FDG-PET/MRI could be beneficial in the early assessment of tumor response to PVL.

Nevertheless, our study has some limitations. The main limitation is the use of normal, healthy liver. In a clinical scenario, the primary and secondary liver tumors usually appear in cirrhotic or fatty livers, in which circumstances the PVL induced alterations and consequently the imaging characteristics of liver lobes may vary. Furthermore, this study examined only tumor-free animals, however, the altered circumstances caused by PVL may result in alteration of tumor FDG uptake as well. Further research on tumor-bearing animals is needed to evaluate this potential tumor response regarding to FDG-uptake after PVL. Finally, it should be noted that FDG is appropriate for the diagnosis only of FDG positive tumors. However, some liver cancer, such as hepatocellular carcinoma is characterized by variable FDG uptake caused by the variation in the expression of G-6Pase. In such case, the results from FDG-PET/MRI could become uncertain, therefore more specific tracers, such as [^11^C]-acetate should be used. [40].

## Conclusion

The aim of the study was to evaluate the FDG uptake pattern in healthy liver after PVL in small animals. Our result showed an altered FDG uptake pattern in both ligated and non-ligated liver lobes after PVL. These alterations in FDG uptake should be taken into account during the assessment of PET data until the PVL induced atrophic and regenerative processes are completed. Because this initial study merely examined healthy animals, further preclinical and clinical studies are needed to define the impact of FDG-PET in therapeutic follow-up after a two stage-hepatectomy combined with PVL.

## References

[1] Kawasaki S, Makuuchi M, Kakazu T, Miyagawa S, Takayama T, et al (1994) Resection for multiple metastatic liver tumors after portal embolization. Surgery 115: 674–677. 8197557

[2] Takayama T (2011) Surgical treatment for hepatocellular carcinoma. Jpn J Clin Oncol 41: 447–454. 21411469 10.1093/jjco/hyr016

[3] Bengmark S, Ekberg H, Evander A, Klofver-Stahl B, Tranberg KG (1988) Major liver resection for hilar cholangiocarcinoma. Ann Surg 207: 120–125. 2829760 10.1097/00000658-198802000-00002 PMC1493364

[4] Gock M, Eipel C, Linnebacher M, Klar E, Vollmar B (2011) Impact of portal branch ligation on tissue regeneration, microcirculatory response and microarchitecture in portal blood-deprived and undeprived liver tissue. Microvasc Res 81: 274–280. 21397614 10.1016/j.mvr.2011.03.005

[5] Rozga J, Jeppsson B, Bengmark S (1986) Portal branch ligation in the rat. Reevaluation of a model. Am J Pathol 125: 300–308. 3789089 PMC1888241

[6] Broering DC, Hillert C, Krupski G, Fischer L, Mueller L, et al (2002) Portal vein embolization vs. portal vein ligation for induction of hypertrophy of the future liver remnant. J Gastrointest Surg 6: 905–913 discussion 913. 12504230 10.1016/s1091-255x(02)00122-1

[7] Clavien PA, Petrowsky H, DeOliveira ML, Graf R (2007) Strategies for safer liver surgery and partial liver transplantation. N Engl J Med 356: 1545–1559. 17429086 10.1056/NEJMra065156

[8] Kokudo N, Tada K, Seki M, Ohta H, Azekura K, et al (2001) Proliferative activity of intrahepatic colorectal metastases after preoperative hemihepatic portal vein embolization. Hepatology 34: 267–272. 11481611 10.1053/jhep.2001.26513

[9] Barbaro B, Di Stasi C, Nuzzo G, Vellone M, Giuliante F, et al (2003) Preoperative right portal vein embolization in patients with metastatic liver disease. Metastatic liver volumes after RPVE. Acta Radiol 44: 98–102. 12631007

[10] Heinrich S, Jochum W, Graf R, Clavien PA (2006) Portal vein ligation and partial hepatectomy differentially influence growth of intrahepatic metastasis and liver regeneration in mice. J Hepatol 45: 35–42. 16698111 10.1016/j.jhep.2006.02.020

[11] Elias D, De Baere T, Roche A, Mducreux, Leclere J, et al (1999) During liver regeneration following right portal embolization the growth rate of liver metastases is more rapid than that of the liver parenchyma. Br J Surg 86: 784–788. 10383579 10.1046/j.1365-2168.1999.01154.x

[12] de Graaf W, van den Esschert JW, van Lienden KP, van Gulik TM (2009) Induction of tumor growth after preoperative portal vein embolization: is it a real problem? Ann Surg Oncol 16: 423–430. 19050974 10.1245/s10434-008-0222-6

[13] Hahn O, Dudas I, Pajor P, Gyorke T, Korom C, et al (2013) [ALPPS (Associated Liver Partition and Portal vein ligation for Staged hepatectomy) – faster and greater growth of liver]. Magy Seb 66: 21–26. 23428724 10.1556/MaSeb.66.2013.1.3

[14] Abdalla EK, Hicks ME, Vauthey JN (2001) Portal vein embolization: rationale, technique and future prospects. Br J Surg 88: 165–175. 11167863 10.1046/j.1365-2168.2001.01658.x

[15] de Graaf W, van Lienden KP, van den Esschert JW, Bennink RJ, van Gulik TM (2011) Increase in future remnant liver function after preoperative portal vein embolization. Br J Surg 98: 825–834. 21484773 10.1002/bjs.7456

[16] Hammerstingl R, Huppertz A, Breuer J, Balzer T, Blakeborough A, et al (2008) Diagnostic efficacy of gadoxetic acid (Primovist)-enhanced MRI and spiral CT for a therapeutic strategy: comparison with intraoperative and histopathologic findings in focal liver lesions. Eur Radiol 18: 457–467. 18058107 10.1007/s00330-007-0716-9

[17] Willatt JM, Hussain HK, Adusumilli S, Marrero JA (2008) MR Imaging of hepatocellular carcinoma in the cirrhotic liver: challenges and controversies. Radiology 247: 311–330. 18430871 10.1148/radiol.2472061331

[18] Juweid ME, Cheson BD (2006) Positron-emission tomography and assessment of cancer therapy. N Engl J Med 354: 496–507. 16452561 10.1056/NEJMra050276

[19] Antoch G, Vogt FM, Veit P, Freudenberg LS, Blechschmid N, et al (2005) Assessment of liver tissue after radiofrequency ablation: findings with different imaging procedures. J Nucl Med 46: 520–525. 15750168

[20] Vogt FM, Antoch G, Veit P, Freudenberg LS, Blechschmid N, et al (2007) Morphologic and functional changes in nontumorous liver tissue after radiofrequency ablation in an in vivo model: comparison of 18F-FDG PET/CT, MRI, ultrasound, and CT. J Nucl Med 48: 1836–1844. 17942811 10.2967/jnumed.107.042846

[21] Bennink RJ, Dinant S, Erdogan D, Heijnen BH, Straatsburg IH, et al (2004) Preoperative assessment of postoperative remnant liver function using hepatobiliary scintigraphy. J Nucl Med 45: 965–971. 15181131

[22] Suzuki S, Toledo-Pereyra LH, Rodriguez FJ, Cejalvo D (1993) Neutrophil infiltration as an important factor in liver ischemia and reperfusion injury. Modulating effects of FK506 and cyclosporine. Transplantation 55: 1265–1272. 7685932 10.1097/00007890-199306000-00011

[23] Hsiao IT, Lin KJ, Chang SI, Yen TC, Chen TC, et al (2010) Impaired liver regeneration of steatotic rats after portal vein ligation: a particular emphasis on (99m)Tc-DISIDA scintigraphy and adiponectin signaling. J Hepatol 52: 540–549. 20206399 10.1016/j.jhep.2010.01.005

[24] Abdalla EK, Barnett CC, Doherty D, Curley SA, Vauthey JN (2002) Extended hepatectomy in patients with hepatobiliary malignancies with and without preoperative portal vein embolization. Arch Surg 137: 675–680 discussion 680–671. 12049538 10.1001/archsurg.137.6.675

[25] Kubota K, Ishiwata K, Kubota R, Yamada S, Tada M, et al (1991) Tracer feasibility for monitoring tumor radiotherapy: a quadruple tracer study with fluorine-18-fluorodeoxyglucose or fluorine-18-fluorodeoxyuridine, L-[methyl-14C]methionine, [6-3H]thymidine, and gallium-67. J Nucl Med 32: 2118–2123. 1834814

[26] Ikeda K, Kinoshita H, Hirohashi K, Kubo S, Kaneda K (1995) The ultrastructure, kinetics and intralobular distribution of apoptotic hepatocytes after portal branch ligation with special reference to their relationship to necrotic hepatocytes. Arch Histol Cytol 58: 171–184. 7576869 10.1679/aohc.58.171

[27] Stewart EE, Chen X, Hadway J, Lee TY (2006) Correlation between hepatic tumor blood flow and glucose utilization in a rabbit liver tumor model. Radiology 239: 740–750. 16621929 10.1148/radiol.2393041382

[28] Song S, Xiong C, Lu W, Ku G, Huang G, et al (2013) Apoptosis imaging probe predicts early chemotherapy response in preclinical models: A comparative study with 18F-FDG PET. J Nucl Med 54: 104–110. 23283564 10.2967/jnumed.112.109397

[29] Takei T, Kuge Y, Zhao S, Sato M, Strauss HW, et al (2005) Enhanced apoptotic reaction correlates with suppressed tumor glucose utilization after cytotoxic chemotherapy: use of 99mTc-Annexin V, 18F-FDG, and histologic evaluation. J Nucl Med 46: 794–799. 15872353

[30] Abouzied MM, Crawford ES, Nabi HA (2005) 18F-FDG imaging: pitfalls and artifacts. J Nucl Med Technol 33: 145–155; quiz 162–143. 16145222

[31] Caraco C, Aloj L, Chen LY, Chou JY, Eckelman WC (2000) Cellular release of [18F]2-fluoro-2-deoxyglucose as a function of the glucose-6-phosphatase enzyme system. J Biol Chem 275: 18489–18494. 10764804 10.1074/jbc.M908096199

[32] Mueller L, Grotelueschen R, Meyer J, Vashist YK, Abdulgawad A, et al (2003) Sustained function in atrophying liver tissue after portal branch ligation in the rat. J Surg Res 114: 146–155. 14559440 10.1016/s0022-4804(03)00252-x

[33] Zakko WF, Berg CL, Gollan JL, Green RM (1998) Hepatocellular expression of glucose-6-phosphatase is unaltered during hepatic regeneration. Am J Physiol 275: G717–722. 9756502 10.1152/ajpgi.1998.275.4.G717

[34] Pauwels EK, Ribeiro MJ, Stoot JH, McCready VR, Bourguignon M, et al (1998) FDG accumulation and tumor biology. Nucl Med Biol 25: 317–322. 9639291 10.1016/s0969-8051(97)00226-6

[35] Rosa JL, Perez JX, Detheux M, Van Schaftingen E, Bartrons R (1997) Gene expression of glucokinase regulatory protein in regenerating rat liver. Hepatology 25: 324–328. 9021942 10.1002/hep.510250212

[36] Brinkmann A, Katz N, Sasse D, Jungermann K (1978) Increase of the gluconeogenic and decrease of the glycolytic capacity of rat liver with a change of the metabolic zonation after partial hepatectomy. Hoppe Seylers Z Physiol Chem 359: 1561–1571. 215501 10.1515/bchm2.1978.359.2.1561

[37] Rocheleau B, Ethier C, Houle R, Huet PM, Bilodeau M (1999) Hepatic artery buffer response following left portal vein ligation: its role in liver tissue homeostasis. Am J Physiol 277: G1000–1007. 10564106 10.1152/ajpgi.1999.277.5.G1000

[38] Alexander B, Rogers C, Naftalin R (2002) Hepatic arterial perfusion decreases intrahepatic shunting and maintains glucose uptake in the rat liver. Pflugers Arch 444: 291–298. 11976943 10.1007/s00424-002-0815-z

[39] Pauwels EK, Sturm EJ, Bombardieri E, Cleton FJ, Stokkel MP (2000) Positron-emission tomography with [18F]fluorodeoxyglucose. Part I. Biochemical uptake mechanism and its implication for clinical studies. J Cancer Res Clin Oncol 126: 549–559. 11043392 10.1007/PL00008465 PMC12165134

[40] Ho CL, Yu SC, Yeung DW (2003) 11C-acetate PET imaging in hepatocellular carcinoma and other liver masses. J Nucl Med 44: 213–221. 12571212

